# Supplementary figures and images for: Ancestry informative alleles captured with reduced representation library sequencing in Theobroma cacao
Source: PLoS One. 2018 Oct 17;13(10):e0203973. doi: 10.1371/journal.pone.0203973 (PMC6192562; doi:10.1371/journal.pone.0203973)

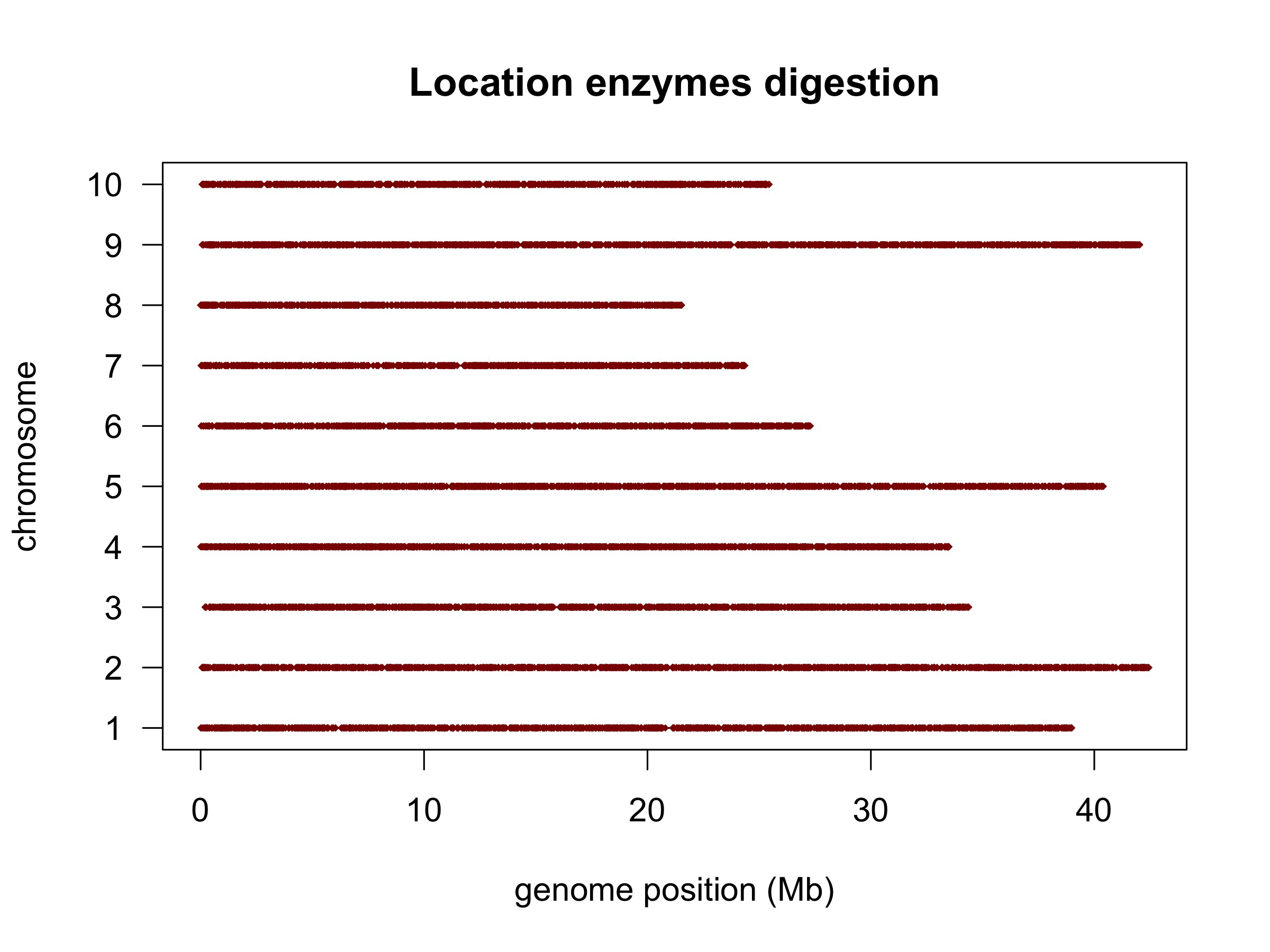

Supplement: S1 Fig — (TIF) [file pone.0203973.s003.tif]

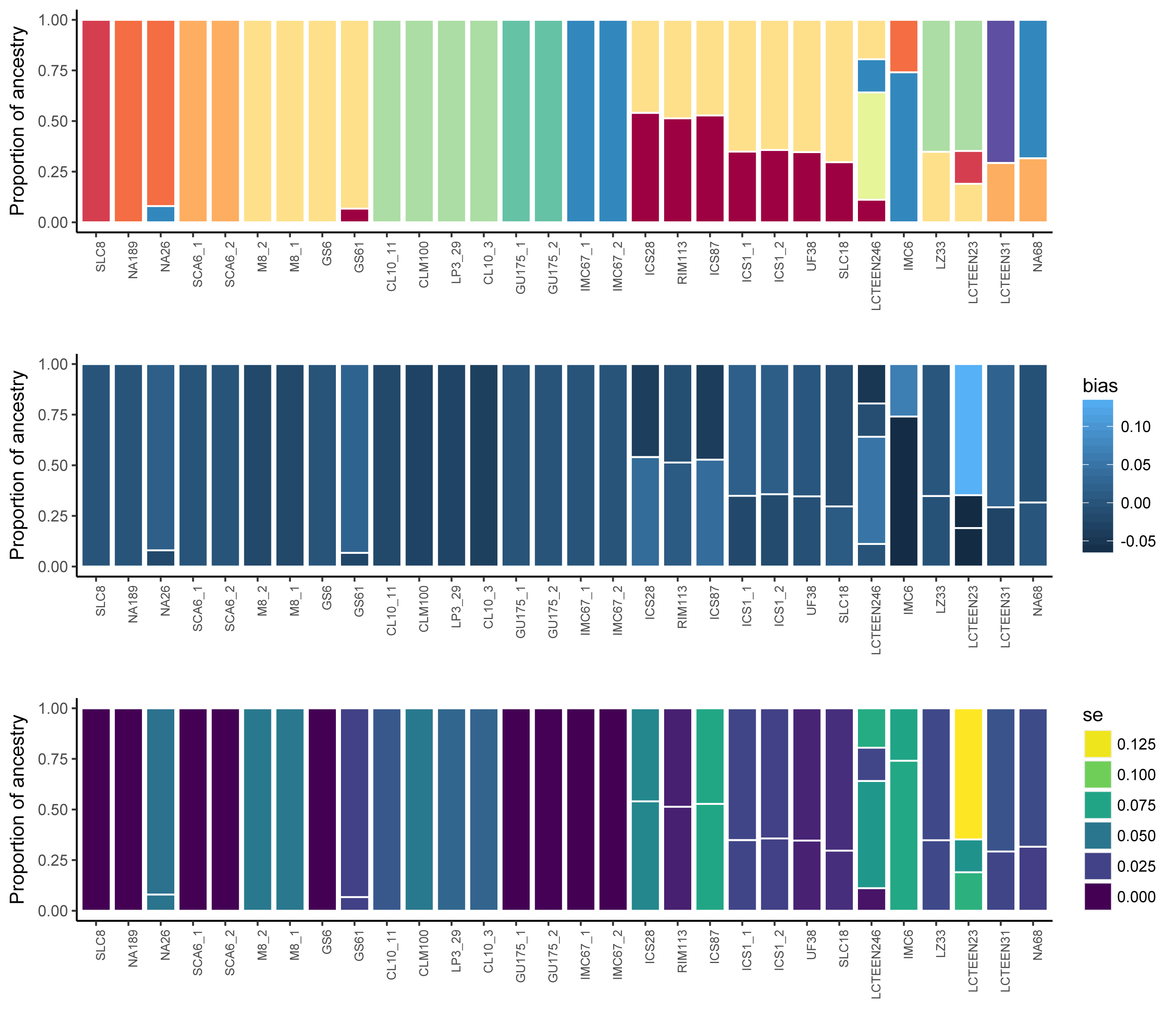

Supplement: S2 Fig — (A) ADMIXTURE bar plot, (B) bias bar plot and (C) standard error for each ancestry. (TIF) [file pone.0203973.s004.tif]
